# Identification of Immune-Related Prognostic Biomarkers Based on the Tumor Microenvironment in 20 Malignant Tumor Types With Poor Prognosis

**DOI:** 10.3389/fonc.2020.01008

**Published:** 2020-07-31

**Authors:** Yu Liu, Hao Zhou, Ji Zheng, Xiaojun Zeng, Wenjing Yu, Wei Liu, Guorong Huang, Yang Zhang, Weiling Fu

**Affiliations:** ^1^Department of Laboratory Medicine, First Affiliated Hospital, Third Military Medical University (Army Medical University), Chongqing, China; ^2^Department of Urology, First Affiliated Hospital, Third Military Medical University (Army Medical University), Chongqing, China; ^3^Department of Laboratory Medicine, Chongqing University Cancer Hospital, Chongqing, China

**Keywords:** prognostic biomarkers, tumor microenvironment, stromal score, immune score, TCGA

## Abstract

Cancer, especially malignant tumors with poor prognosis, has become a major hazard to human life and health. The tumor microenvironment is gaining increasing attention from researchers, as it offers a new focus for tumor diagnosis, therapy, and prognosis. The numbers of immune and stromal cells, which are major components of the tumor microenvironment, could be determined from RNA-seq data with the Estimation of STromal and Immune cells in Malignant Tumors using Expression data (ESTIMATE) algorithm. To explore the effects of immune and stromal cells on tumor prognosis, we analyzed associations between overall survival and immune/stromal scores for 20 malignant tumor types based on The Cancer Genome Atlas (TCGA) data. For six of the 20 tumor types, we observed statistically significant associations. Furthermore, to better explain the predictive ability of these scores, differentially expressed genes (DEGs) were identified in groups of cases with high or low immune or stromal scores for each of these six malignant tumor types. In addition, a list of immune-related genes was screened to identify prognostic predictors for one or more tumor types. Thus, multi-database joint analysis can provide a new approach to the assessment of tumor prognosis and allow the identification of related genes that may be new biomarkers for tumor metastasis and prognosis.

## Introduction

Currently, cancer is a serious public health problem due to its serious threat to human life and health. According to the latest statistics, over the past decade, the overall cancer incidence rate has remained generally stable in women and decreased in men, but cancer death rates in both sexes have declined annually due to behavioral changes and medical practices, such as cancer screening tests (1). To gain insight into the occurrence and progression of cancers through molecular characterization, comprehensive genomic data resources have been established for the broad research community, including the UCSC Genome Browser and The Cancer Genome Atlas (TCGA) (2, 3). TCGA provides rich multi-omic data for 33 malignant tumor types with both good and poor prognosis, allowing convenient application in the computational biology field.

Malignant tumor tissues not only contain tumor cells but also require support from tumor-associated normal cells, including stromal cells and infiltrating immune cells, which together comprise the tumor microenvironment (4, 5). A growing body of evidence has demonstrated the importance of the microenvironment for tumor initiation, progression, and even therapeutic approaches (6, 7). Thus, understanding the tumor microenvironment has received a new emphasis on tumor biology, therapy, and prediction. However, the presence of normal cells in samples strongly affects the observed levels of gene expression, leading to significantly lower accuracy of tumor cell gene expression profiles (8). As a result, many methods have been established to infer tumor purity using gene expression data (9–11).

ESTIMATE (Estimation of STromal and Immune cells in Malignant Tumors using Expression data) is an algorithm that uses the gene expression signatures of tumor samples from TCGA to infer tumor purity (11). As the most common normal cells in the microenvironment, both stromal and immune cells were chosen for evaluation in this study, and their gene expression data were used to calculate stromal and immune scores to estimate the numbers of infiltrating stromal cells and immune cells in tumor samples. In addition, by combining the stromal and immune scores, an ESTIMATE score can also be calculated to characterize tumor purity. At present, approximately 25 malignant tumor types have been evaluated by ESTIMATE using expression data across different platforms (12). Furthermore, stromal or immune scores have been analyzed in clinical data to predict tumor progression (13), treatment (14), and prognosis (15). However, these investigations involve only one cancer and therefore cannot compare the differences and explore common characteristics among many kinds of cancers.

In this study, we investigated the correlation between prognosis and stromal and immune scores in cases of 20 malignant tumor types to explore the prognostic potential of the tumor microenvironment, using both TCGA and the ESTIMATE algorithm, for the first time. Finally, six malignant tumor types with poor prognosis, including breast invasive carcinoma (BRCA), lung adenocarcinoma (LUAD), kidney renal clear cell carcinoma (KIRC), stomach adenocarcinoma (STAD), brain lower grade glioma (LGG) and skin cutaneous melanoma (SKCM), were screened for prognostic evaluation using stromal or immune scores. On this basis, a list of microenvironment-associated differentially expressed genes (DEGs) was extracted and further analyzed via Gene Ontology (GO), protein-protein interaction (PPI), survival and expression analysis to effectively identify genes as potential prognostic biomarkers for each kind of tumor and even for multiple tumor types.

## Materials and Methods

### Selection of Tumor Types for the Analysis

Twenty malignant tumor types were chosen based on the data integrity, sample size, and overlap between TCGA and ESTIMATE. TCGA datasets for bladder urothelial carcinoma (BLCA, number of samples: *n* = 436), BRCA (*n* = 1247), cervical cancer (CESC, *n* = 312), colorectal adenocarcinoma (COAD, *n* = 551), esophageal carcinoma (ESCA, *n* = 204), glioblastoma multiforme (GBM, *n* = 629), head and neck squamous cell carcinoma (HNSC, *n* = 604), KIRC (*n* = 945), kidney renal papillary cell carcinoma (KIRP, *n* = 352), liver hepatocellular carcinoma (LIHC, *n* = 438), brain LGG (*n* = 530), LUAD (*n* = 706), lung squamous cell carcinoma (LUSC, *n* = 626), SKCM (*n* = 481), ovarian serous cystadenocarcinoma (OV, *n* = 630), pancreatic ductal adenocarcinoma (PAAD, *n* = 196), pheochromocytoma & paraganglioma (PCPG, *n* = 187), prostate adenocarcinoma (PRAD, *n* = 566), STAD (*n* = 580), and thyroid papillary carcinoma (THCA, *n* = 580) were selected, and the “Phenotype” information for each dataset was downloaded for the survival analyses using the UCSC Xena Browser portal (16) (https://xenabrowser.net/datapages/). The immune scores and stromal scores for the human cancers above were downloaded from the ESTIMATE website (11) (https://bioinformatics.mdanderson.org/estimate/). RNA-seq gene expression data from the Illumina HiSeq 2000 RNA Sequencing platform for BRCA, LUAD, KIRC, STAD, LGG and SKCM were also obtained from the TCGA data portal.

### Kaplan-Meier Curves Based on High/Low Stromal or Immune Scores

The cases of each cancer, including the stromal and immune score values and the overall survival in the “Phenotype” file, were chosen and matched with each other. The values of the immune scores and stromal scores were sorted from low to high and divided in half to form the low and high score groups for the cases of each cancer. Then, Kaplan-Meier survival curves were plotted to demonstrate the correlation between the patients' overall survival and the low and high immune and stromal score groups for the 20 malignant tumor types with a log-rank test. Simultaneously, the median survival time (MST), hazard ratio with a 95% confidence interval (CI) and *p*-value were calculated and analyzed.

### Identification of DEGs for Six Malignant Tumor Types

For BRCA, LUAD, KIRC, STAD, LGG and SKCM, DEGs between the low and high immune or stromal score groups were identified by the limma algorithm (17) online (http://www.omicsbean.cn/) with fold change (FC) value > 1.5 and adjusted *p*-value < 0.05. Venn diagrams were used to obtain the common DEGs for two groups: the immune score group (BRCA, LUAD, KIRC, LGG and SKCM cases; tumor types correlated with immune scores) and the stromal score group (STAD, LGG and SKCM cases; tumor types correlated with stromal scores). TBtools software was used to display the expression profiles of the top 100 DEGs in the form of a heatmap (18).

### Functional Enrichment Analyses of Common DEGs

GO (19) and Kyoto Encyclopedia of Genes and Genomes (KEGG) (20) are the two most important databases traditionally used for gene list enrichment analyses. DAVID (21) (Functional Annotation Bioinformatics Microarray Analysis) (https://david.ncifcrf.gov/), an online bioinformatics tool, was used to present the DEG enrichment of the GO biological process (BP), cellular component (CC), and molecular function (MF) terms and KEGG pathways with a false discovery rate (FDR) < 0.05.

### PPI Network and Module Analysis

PPI information was evaluated by STRING version 11.0 (22) (https://string-db.org/), an online tool. Then, Cytoscape software (23) was applied to reconstruct and analyze the PPI network. The modules of the PPI network were further checked by the MCODE (Molecular Complex Detection) app in Cytoscape software, which found densely connected regions using the following parameters: degree cutoff = 2, k-core = 2, max. depth = 100 and node score cutoff = 0.2.

### Survival Analysis and Gene Expression Analysis

The common DEGs from the top list for immune scores analysis and top list for stromal scores analysis were carried out by survival analysis through TCGA analysis in the UALCAN cancer database (http://ualcan.path.uab.edu/) (24). Based on the gene expression in the corresponding tumor, the UALCAN cancer database could provide Kaplan-Meier survival curves and *p*-values to obtain DEGs related to prognosis in associated cancers with statistically significant differences (*p* < 0.01). Next, the expression of the top DEGs related to prognosis was validated in tumor and normal samples using the Gene Expression Profiling Interactive Analysis (GEPIA) database (http://gepia.cancer-pku.cn/) (25). Based on tumor and normal samples from the TCGA and GTEx projects, GEPIA was used to analyze differential gene expression in tumors with *p*-value < 0.01 and |log_2_FC|>1.

## Results

### Correlation of Prognosis With Stromal and Immune Scores in 20 Different Tumor Types

**Figure 1:**
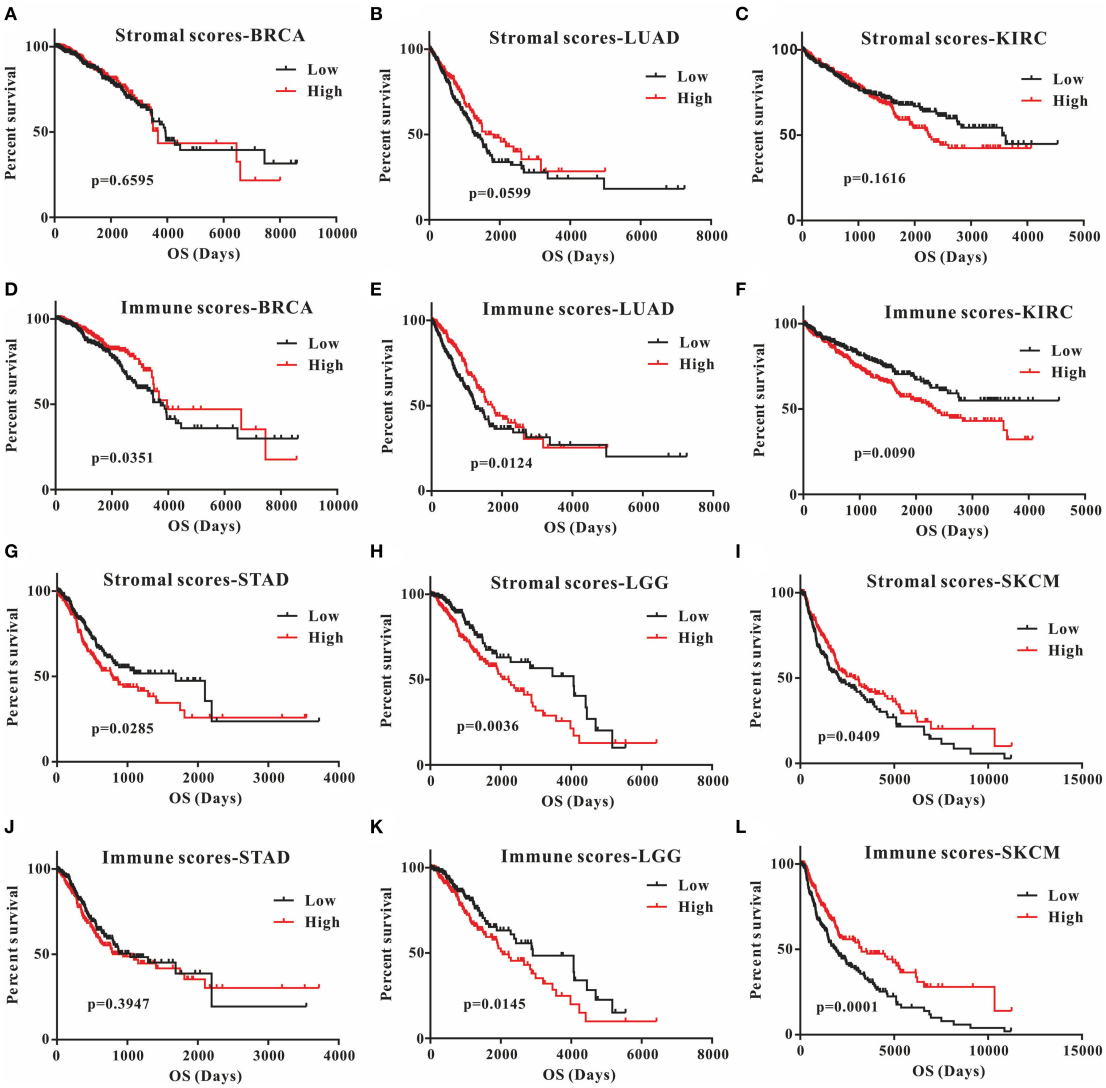
Correlation of stromal scores and immune scores with prognosis in six different tumor types by Kaplan-Meier survival curves. **(A–C,G–I)** Kaplan-Meier survival curves for BRCA, LUAD, KIRC, STAD, LGG, and SKCM grouped by stromal scores. **(D–F, J–L)** Kaplan-Meier survival curves for BRCA, LUAD, KIRC, STAD, LGG, and SKCM grouped by immune scores. Low, low score group (black line); high, high score group (red line).

This was the first study to investigate whether stromal and immune scores could predict prognosis for 20 different tumor types. The correlations between overall survival and stromal or immune scores are shown in Table 1. For BRCA, LUAD and KIRC, the immune scores were more informative than the stromal scores to predict prognosis, because there were statistically significant differences in overall survival between the low and high immune score groups (*p* < 0.05) (Figures 1A–F). In contrast, for STAD, a significant difference in stromal scores was observed (*p* = 0.0285) (Figures 1G,J). Moreover, statistically significant differences were found in both the stromal and immune scores for LGG and SKCM, indicating a high potential for prognostic evaluation (Figures 1H,I,K,L). However, for the other 14 tumor types, there were no statistically significant differences using the stromal and immune score groups (*p* > 0.05).

**Table 1:** Correlation analysis between overall survival and stromal scores or immune scores in 20 different tumor types.

**Cancer type**	**Low N**	**High N**	**Stromal score**				**Immune score**			
			**Low MST**	**High MST**	**HR (95% CI)**	***p*-Value^#^**	**Low MST**	**High MST**	**HR (95% CI)**	***p*-Value^#^**
BLCA	202	202	1,348	859	0.77 (0.58–1.04)	0.0864	1,036	1,005	1.02 (0.76–1.37)	0.8877
BRCA	542	543	3,941	3,669	1.07 (0.78–1.48)	0.2648	3,736	3,959	1.41 (1.03–1.93)	0.0351^*^
CESC	146	147	3,046	3,097	1.16 (0.73–1.84)	0.5291	4,086	3046	1.50 (0.94–2.37)	0.0886
COAD	124	125	Undefined	2,134	1.02 (0.56–1.86)	0.9391	Undefined	2,134	1.00 (0.55–1.81)	0.9956
ESCA	92	93	855	681	0.85 (0.54–1.33)	0.4791	801	694	0.86 (0.55–1.35)	0.5168
GBM	82	82	442	380	0.84 (0.58–1.17)	0.2883^*^	427	375	0.92 (0.65–1.31)	0.6488
HNSC	259	260	1,504	1,838	1.03 (0.79–1.34)	0.8338	1,430	1,762	1.21 (0.93–1.58)	0.1615
KIRC	266	266	3,554	2,256	0.81 (0.60–1.09)	0.1616	Undefined	2,343	0.67 (0.50–0.91)	0.0090^**^
KIRP	144	144	Undefined	2,941	0.70 (0.39–1.26)	0.2306	Undefined	Undefined	1.26 (0.70–2.28)	0.4408
LIHC	183	184	2,486	1,694	1.14 (0.81–1.61)	0.4583	1,791	1,685	1.13 (0.80–1.59)	0.4990
LGG	262	263	4,068	2,235	0.60 (0.43–0.85)	0.0036^**^	2,907	2,052	0.65 (0.46–0.92)	0.0145^*^
LUAD	252	253	1,293	1,830	1.32 (0.99–1.77)	0.0599	1,235	1,725	1.45 (1.09–1.94)	0.0124^*^
LUSC	247	247	1,695	1,470	0.88 (0.68–1.16)	0.3524	1,695	1,655	0.95 (0.72–1.26)	0.7398
SKCM	230	230	2,030	2,927	1.31 (1.02–1.73)	0.0409^*^	1,860	3,259	1.69 (1.30–2.21)	0.0001^***^
OV	231	232	1,359	1,324	0.94 (0.75–1.18)	0.5784	1,334	1,399	1.09 (0.87–1.37)	0.4474
PAAD	89	89	634	603	0.92 (0.61– 1.37)	0.6703	627	603	1.01 (0.67–1.51)	0.9801
PCPG	91	91	Undefined	Undefined	2.94 (0.67–10.78)	0.1632	Undefined	Undefined	1.41 (0.35–5.70)	0.6291
PRAD	248	249	Undefined	Undefined	2.20 (0.65–7.73)	0.2221	Undefined	Undefined	1.39 (0.40–4.81)	0.6035
STAD	194	194	1,686	794	0.70 (0.51–0.96)	0.0285^*^	1,043	940	0.87 (0.65–1.19)	0.3947
THCA	254	254	Undefined	Undefined	1.03 (0.39–2.74)	0.9527	Undefined	Undefined	1.00 (0.37–2.66)	0.9962

# *p-values and HR were calculated by applying the log-rank test; N, number; MST, median survival time; HR, hazard ratio; undefined, the cumulative survival was not <50%*;

* *p-value < 0.05*;

** *p-value < 0.01*;

*** *p-value < 0.001*.

Although stromal scores or immune scores were significantly correlated with prognosis for BRCA, LUAD, KIRC, STAD, LGG, and SKCM, it is necessary to further research how low and high scores can estimate prognosis. For BRCA, LUAD and KIRC, which were correlated with immune scores, the MST of BRCA (3,959 vs. 3,736 d, high vs. low) and LUAD (1,725 vs. 1,235 d) cases were longer in the high score group than in the low score group, but the opposite result was found for KIRC cases (2,343 vs. > 4,000 d). The MST of STAD cases with low stromal scores was longer than that of the cases in the high score group (794 vs. 1,686 d). Moreover, for LGG, the MST in the low score group was longer than that in the high score group for both stromal (2,235 vs. 4,068 d) and immune scores (2,052 vs. 2,907 d). However, the opposite results were observed for SKCM cases, which were also correlated with both stromal (2,927 vs. 2,030 d) and immune scores (3,259 vs. 1,860 d). Overall, these results suggest that stromal scores or immune scores can help to evaluate prognosis for 6 malignant tumor types with poor prognosis.

### Identification of DEGs Associated With High Stromal and Immune Scores

**Figure 2:**
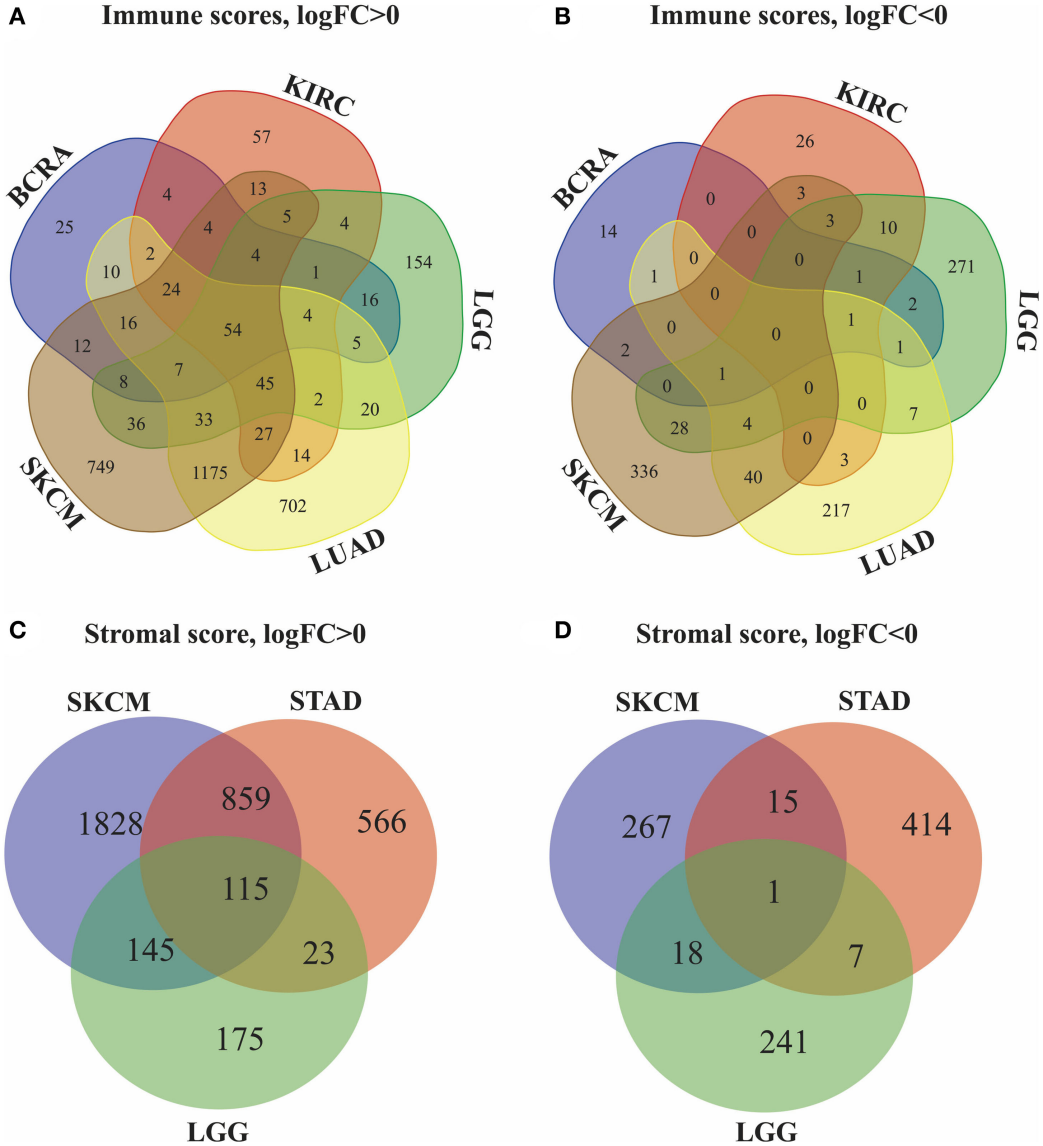
Identification of DEGs in the stromal and immune score groups by Venn diagrams. **(A)** Upregulated DEGs identified through immune scores; **(B)** downregulated DEGs identified through immune scores; **(C)** upregulated DEGs identified through stromal scores; **(D)** downregulated DEGs identified through stromal scores.

Next, we identified genes whose expression was positively or negatively associated with immune or stromal score values. For this analysis, we selected six tumors whose prognosis could be predicted by immune (BRCA, LUAD, KIRC, LGG, and SKCM) and/or stromal (STAD, LGG, and SKCM) scores. Then, Venn diagrams were drawn to show the common upregulated and downregulated genes (Figures 2A,B). The results revealed a total of 54 common DEGs among the upregulated genes, but no common DEGs were detected among the downregulated genes. In addition, among these 5 cancers (BRCA, LUAD, KIRC, LGG and SKCM), the BRCA, LUAD and SKCM cases with long MST and high scores had other 16 common DEGs, and all the DEGs were upregulated. Conversely, for the KIRC and LGG cases with long MST and low scores, 4 and 10 additional upregulated and downregulated common DEGs, respectively, were detected (Table S1).

Because stromal scores were correlated with prognosis for STAD, LGG and SKCM, the common upregulated and downregulated DEGs were examined using Venn diagrams (Figures 2C,D). Among the upregulated genes, a total of 115 common DEGs were detected for STAD, LGG and SKCM, and 23 additional common DEGs were detected for STAD and LGG cases with long MST and low scores. Among the downregulated genes, 1 common DEG was found for STAD, LGG, and SKCM, and 7 additional common DEGs were found for only the STAD and LGG cases (Table S2). Thus, these DEGs were chosen as the focus of all subsequent analyses. What's more, heatmaps showed the expression profiles of the top 100 DEGs that distinguish tumors with low and high immune or stromal scores (Figures S1, S2).

### Go Enrichment Analysis for Common DEGs

**Figure 3:**
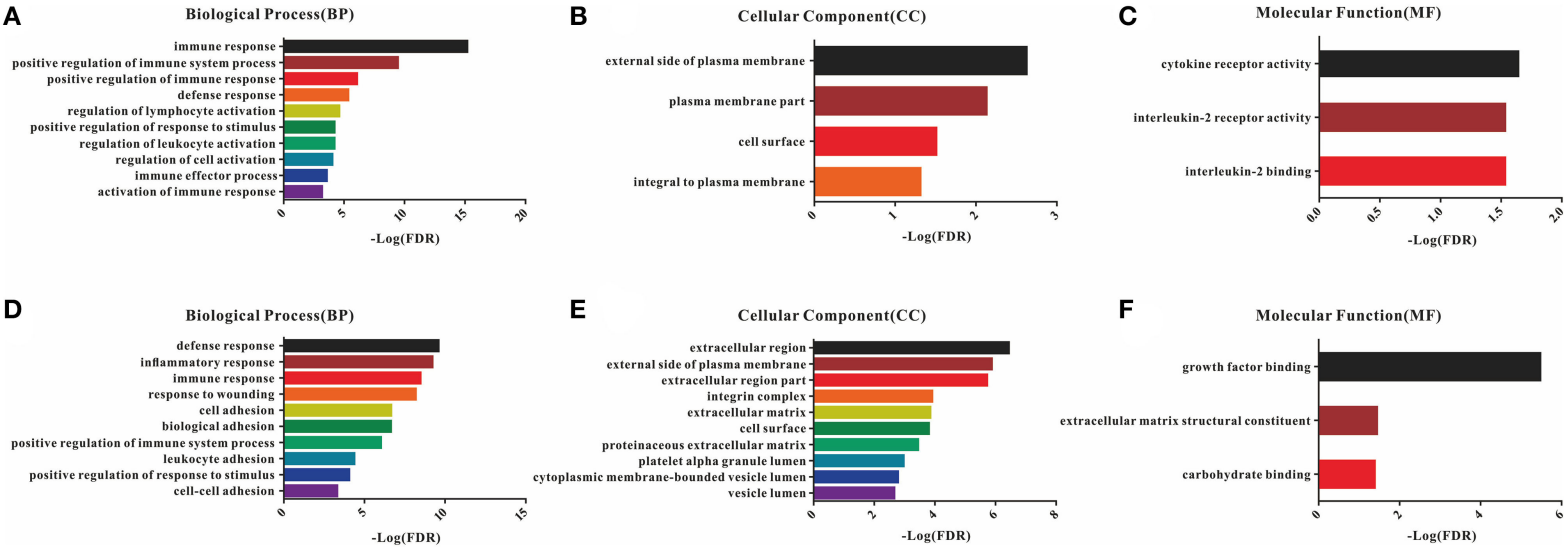
GO enrichment analysis of DEGs detected 54 in the immune score group and 116 in the stromal score group. **(A–C)** Top 10 GO terms in BP, CC, and MF for the immune score group; **(D–F)** top 10 GO terms in BP, CC, and MF for the stromal score group.

A total of 54 common DEGs detected in the immune score group and 116 common DEGs detected in the stromal score group were chosen for functional enrichment analyses. First, the results of the gene enrichment analyses are shown in Figure 3, including the three GO categories BP, CC and MF. There were 28 enriched terms in BP (the top 10 are shown), four terms in CC and three terms in MF with FDR < 0.05 for the immune score group (Figures 3A–C), and 21 BP terms, 16 CC terms and 3 MF terms for the stromal score group (Figures 3D–F).

In the BP category, most DEGs were associated with the immune process, including immune response, immune system process, defense response, and inflammatory response for both the immune and stromal score groups (Table S3). Plasma membrane terms dominated the CC category, representing 40.74 and 44.83% of the DEGs in the immune and stromal score groups, respectively. Finally, the DEGs were clustered based on the MF category, and the results showed that a majority of the genes were associated with receptor activity and binding reaction. A comparison showed a high consistency of terms in the BP, CC and MF categories in the two different groups. Furthermore, the KEGG pathways for these two groups are shown in Figure S3.

### Comparison of PPI Between Immune and Stromal Score Groups

**Figure 4:**
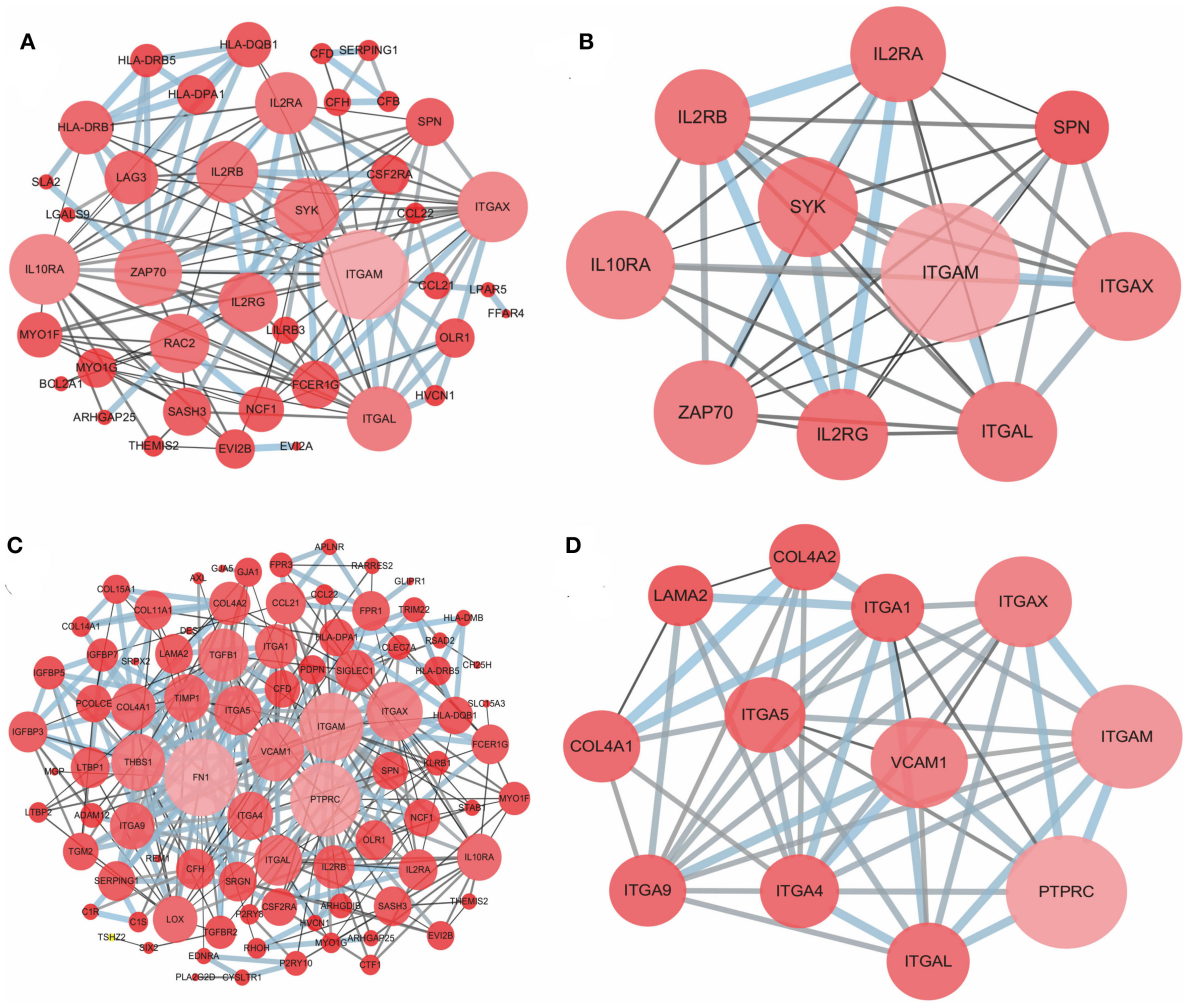
PPI network of common DEGs for the immune and stromal score groups. **(A)** PPI network of 54 DEGs in the immune score group; **(B)** ITGAM module, top 1 PPI network for the immune score group, identified by Cytoscape MCODE; **(C)** PPI network of 116 DEGs in the stromal score group; **(D)** PTPRC module, top 1 PPI network for the stromal score group.

To better understand the interactions of the DEGs in each group and explore the distinction between the immune and stromal score groups, 54 DEGs for the immune score group and 116 DEGs for the stromal score group were analyzed separately using the STRING tool to acquire PPI networks. For the immune score group, the network included 53 nodes and 146 edges with an enrichment *p*-value < 1.0e-16 (Figure 4A). Furthermore, the 10 central nodes were identified by Cytoscape MCODE, all with high degree values, and named the ITGAM module (Figure 4B). For the stromal score group, the network included 115 nodes and 369 edges with an enrichment *p*-value < 1.0e-16 (Figure 4C); 12 central nodes were identified and named the PTPRC module (Figure 4D).

### Prognostic Potential of Each DEG in Associated Cancers

We further investigated the DEGs correlated with prognosis in associated cancers using Kaplan-Meier survival curves from the TCGA database. Among the 54 DEGs for the immune score group, 53 DEGs had expression levels associated with the prognosis of at least one of BRCA, LUAD, KIRC, LGG, and SKCM (*p* < 0.05); only ASGR2 expression was uncorrelated with prognosis (Table 2). In addition, C16orf54 and hepcidin antimicrobial peptide (HAMP) expression levels were both correlated with prognosis in four tumor types, except KIRC and BRCA, and 17 DEGs had expression levels associated with prognosis in three tumor types. For the stromal score group, 108 of the 116 DEGs had expression levels significantly correlated with prognosis in at least one of STAD, LGG, and SKCM (Table S4). Notably, five DEG expression levels were correlated with prognosis in these three tumor types, including AXL, CCDC152, EVI2B, GLIPR1, and SERPING1.

**Table 2:** Correlation of DEGs in the immune score group with prognosis in associated cancers by Kaplan-Meier survival curves from the TCGA database.

	**DEG**	***p* (BRCA)**	***p* (LUAD)**	***p* (KIRC)**	***p* (LGG)**	***p* (SKCM)**
1	ARHGAP25	-	-	-	<0.0001	0.0024
2	BCL2A1	-	-	0.018	0.0053	-
3	C16orf54	0.039	0.038	-	<0.0001	0.0003
4	C1orf38	-	-	0.015	0.032	-
5	CCL21	0.015	-	-	-	-
6	CCL22	-	-	0.018	0.00055	-
7	CFB	-	-	0.017	<0.0001	0.00026
8	CFD	-	-	-	0.0035	0.32
9	CFH	-	-	0.0036	<0.0001	-
10	CLIC2	-	-	0.014	-	0.00028
11	CSF2RA	-	0.02	-	0.0086	-
12	EVI2A	-	0.049	-	-	0.0035
13	EVI2B	-	-	-	<0.0001	0.00011
14	FAM70A	-	-	-	<0.0001	-
15	FCER1G	-	-	0.0021	<0.0001	0.0017
16	GAL3ST4	-	0.012	-	<0.0001	-
17	GPR120	-	-	-	0.0034	0.004
18	HAMP	-	0.0013	0.00018	<0.0001	0.0005
19	HLA-DPA1	-	0.0018	-	<0.0001	<0.0001
20	HLA-DQB1	-	0.016	-	<0.0001	<0.0001
21	HLA-DRB1	-	0.0036	-	<0.0001	<0.0001
22	HLA-DRB5	-	-	-	<0.0001	<0.0001
23	HLA-DRB6	-	-	-	0.00074	<0.0001
24	HVCN1	-	0.042	-	<0.0001	0.031
25	IL10RA	-	-	-	0.017	0.00019
26	IL2RA	-	-	0.003	-	<0.0001
27	IL2RB	-	-		0.00047	0.007
28	IL2RG	-	-	0.047	<0.0001	0.00064
29	ITGAL	-	-	-	<0.0001	0.0014
30	ITGAM	-	-	-	0.0089	0.0002
31	ITGAX	-	-	-	<0.0001	0.0025
32	LAG3	-	-	0.049	0.0014	<0.0001
33	LGALS9	-	-	0.044	<0.0001	0.00028
34	LILRB3	-	-	-	<0.0001	0.002
35	LPAR5	-	0.032	-	-	-
36	MYO1F	-	-	-	0.0014	0.01
37	MYO1G	-	-	-	0.00056	0.00059
38	NCF1	-	-	-	<0.0001	<0.0001
39	OLR1	-	-	-	<0.0001	0.002
40	RAC2	-	-	-	<0.0001	0.0075
41	RARRES3	-	0.0022	-	0.00018	<0.0001
42	RSAD2	-	-	-	<0.0001	0.00012
43	SASH3	-	-	-	<0.0001	-
44	SERPING1	-	-	0.035	<0.0001	0.00043
45	SLA2	-	0.023	-	<0.0001	0.0019
46	SLC37A2	-	0.044	0.014	<0.0001	-
47	SPN	0.0098	-	-	<0.0001	0.0011
48	STAC3	-	-	0.00091	0.00043	0.0019
49	SYK	-	-	-	0.022	-
50	TMEM149	-	-	0.00025	<0.0001	0.022
51	TNFSF12-TNFSF13	-	0.048	-	0.0004	-
52	XAF1	-	-	0.0026	<0.0001	<0.0001
53	ZAP70	-	-	-	<0.0001	0.009

*-, p > 0.05, no statistical significance*.

**Figure 5:**
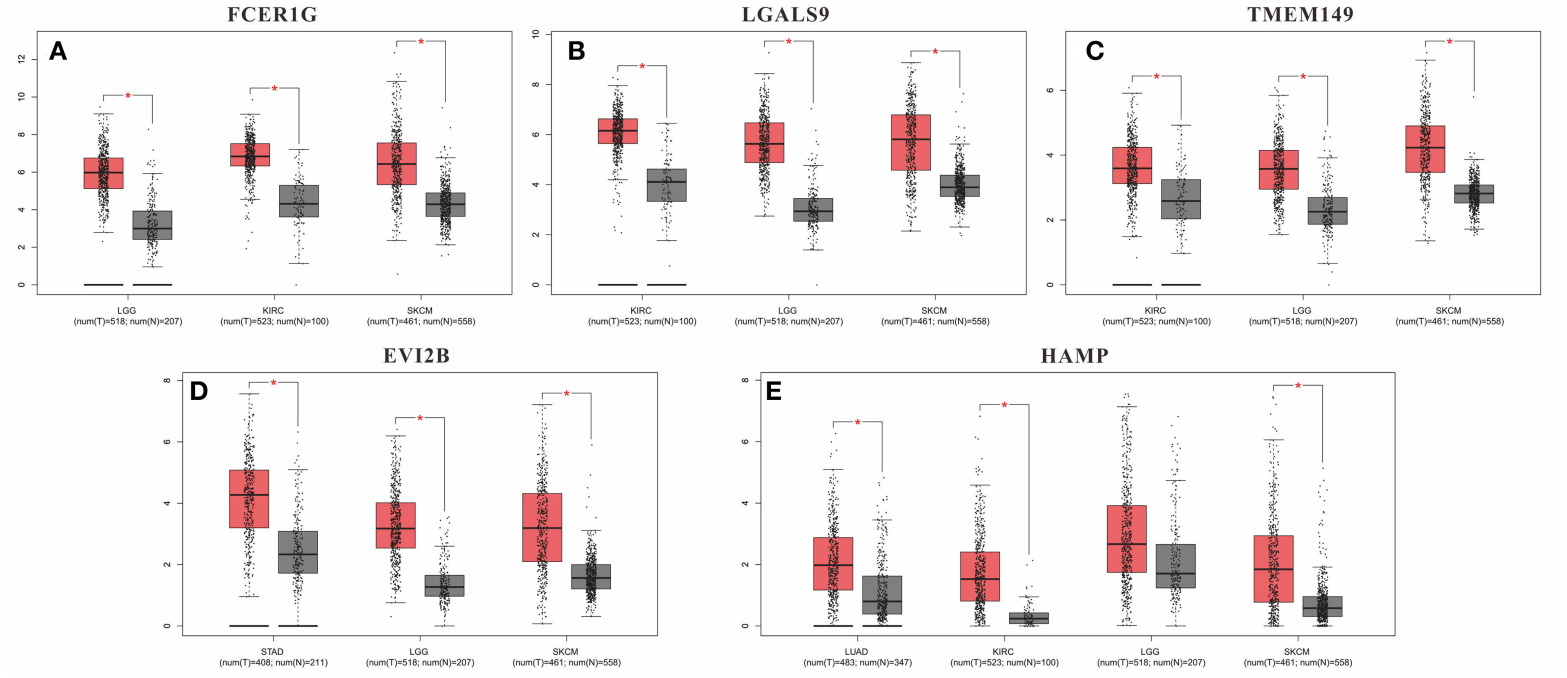
Gene expression levels in the cancer population and normal population. **(A)** FCER1G; **(B)** LGALS9; **(C)** TWEM149; **(D)** EVI2B and **(E)** HAMP. *p-value < 0.05.

Although these DEGs demonstrated prognostic potential in specific cancers, it was necessary to investigate the gene expression differences between the normal and cancer populations. Thus, the 53 DEGs from the immune score group and 108 DEGs from the stromal score group were excavated with GEPIA to identify genes with significantly differential expression. The results revealed that 79 DEGs had statistically significant expression differences (*p* < 0.01), including five genes with differences in all three tumor types, 21 genes with differences in two tumor types, and 53 genes with differences in only one tumor type (Table 3). Moreover, for FCER1G, LGALS9, TWEM149, EVI2B, and HAMP, the gene expression levels in the cancer population were higher than those in the normal population (Figure 5). The expression levels of genes with statistical significance in 2 tumor types are shown in detail in Figure S4.

**Table 3:** Correlation of cancer types with differentially expressed genes between the normal and cancer populations by GEPIA.

**Cancer types**	**DEGs**
KIRC, LGG and SKCM	LGALS9, FCER1G, TMEM149
LUAD, KIRC and SKCM	HAMP
STAD, LGG and SKCM	EVI2B
KIRC and LGG	C1orf38, XAF1, SLC37A2
SKCM and LGG	C1R, FPR3, HLA-DMB, HLA-DPA1, HLA-DRB1, HLA-DRB5, HLA-DRB6, HVCN1, ITGAX, NCF1, RARRES3, SLC15A3, VCAM1
STAD and LGG	DES, FN1, IGFBP7
KIRC and SKCM	IL2RG, LAG3
BRCA	CCL21
KIRC	BCL2A1, CFB, STAC3
LGG	ABI3, APLNR, ARHGDIB, CLEC7A, COL4A1, GAL3ST4, GGT5, IGFBP3, IGFBP5, IL10RA, ITGAM, LTBP1, MFNG, MGP, MRC2, OLR1, PTPRC, RARRES2, TGFB1, TGFBR2, TRIM22, TMEM255A, MYO1F, SASH3, SYK
LUAD	TNFSF12-TNFSF13
SKCM	AXL, CFD, CSF2RA, C1S, EVI2A, ITGAL, IL2RA, PLA2G2D, TMEM97, TIMP1, TGM2, SRGN, REM1, RAC2
STAD	ADAM12, FPR1, FNDC1, GALNTL2, GLIPR1, H19, LAMA2, LOX, STC1

## Discussion

With the development of high-throughput technologies, the omics sciences have advanced greatly, gaining unprecedented development; high-throughput methods have also promoted the progress of bioinformatics by generating thousands of massive datasets called “big data” (26, 27). Thus, data mining has emerged to efficiently transform big data into useful information and knowledge, and several automated tools and techniques are used to intelligently assist data analysis (28, 29). At present, TCGA is the primary database for multi-omic cancer data, and the ESTIMATE algorithm, a new data mining tool, can be used with TCGA data sets to estimate the numbers of stromal and immune cells in the tumor microenvironment and, thus, to assess tumor purity (30).

For the first time, in this study, the correlation of stromal and immune scores with prognosis was investigated for 20 malignant tumor types in an attempt to develop a new prognostic indicator. The results showed that immune scores could predict prognosis for BRCA, LUAD, KIRC, LGG, and SKCM, and stromal scores were significantly correlated with prognosis in STAD, LGG, and SKCM (Figure 1). However, the MST of the BRCA, LUAD, and SKCM cases in the high score group was longer than that in the low score group, and the opposite results were found for the KIRC, LGG and STAD cases (Table 1). These results indicate that stromal or immune scores can act as a new indicator for the above six tumor types, allowing prognosis prediction from a new perspective, that of the tumor microenvironment.

To examine the potential mechanisms and correlations underlying this phenomenon, the gene expression data were used to extract the common DEGs (FC > 1.5 and adjusted *p*-value < 0.05) for the two score groups, and then, the DEGs were further explored by GO enrichment and PPI analysis. As expected, there was a high similarity between the two groups in the BP category, mainly including various terms associated with immune responses, although adhesion responses were also prominent in the stromal score group. Similarly, the CC terms enriched in the DEGs were almost all related to the cell surface in both groups. In the MF category, the DEGs were enriched in cytokine receptor activity, interleukin-2 (IL-2) receptor activity and binding in the immune score group and growth factor binding, extracellular matrix structural constituent and carbohydrate binding in the stromal score group. These results are consistent with a previous report that stromal cells are mainly made up of nonimmune cells, such as endothelial cells and fibroblasts, that function in the extracellular matrix and contribute to the neoplastic phenotype, premalignant progression, tumor invasion and metastasis (31, 32). Nevertheless, immune cells in the tumor microenvironment play a dual role in tumor progression, mainly depending on the associated immune response (33).

Next, the potential associations among the DEGs were confirmed by PPI network analysis. For the immune score group, the 10 central nodes principally involved two types of molecules: integrin and IL receptor (Figure 4B). ITGAM, ITGAX and ITGAL encode the integrin subunit alpha M, X and L chain proteins, respectively; these proteins are the main components of an alpha chain that can be combined with a beta chain to finally form integrin, which mainly functions in cell cycle regulation (34, 35). In addition, among the interacting IL receptor genes, including IL2RA, IL2RB, IL2RG and IL10RA, IL2RA, IL2RB, and IL2RG constitute the high-affinity IL2 receptor, which regulates tolerance and immunity (36). IL10RA encodes the IL10 receptor, which can mediate the immunosuppressive signal of IL10, leading to inhibition of the synthesis of proinflammatory cytokines (37). However, in the stromal score group, seven genes in 12 central nodes encoded integrin, including ITGAM, ITGAX, ITGAL, ITGA1, ITGA4, ITGA5, and ITGA9 (Figure 4D). The most remarkable node was PTPRC, which encodes a member of the protein tyrosine phosphatase family and is involved in the regulation of many cellular processes (38).

Furthermore, we identified whether the common DEGs were correlated with tumor prognosis using Kaplan-Meier survival curves from TCGA, screening 53 and 108 DEGs for the immune and stromal score groups, respectively (Table 2, Table S4). Because a gene must have a measurable difference in expression level to be used as a biomarker, it was necessary to confirm the presence of expression differences between the normal and cancer populations. Finally, there were 79 genes with significant expression differences, and five of these genes were correlated with prognosis in three tumor types simultaneously (Table 3). Moreover, the expression levels of these five genes were higher in tumor tissues than in normal tissues. Thus, LGALS9, FCER1G, and TMEM149 can be regarded as common biomarkers for KIRC, LGG, and SKCM. EVI2B is a common biomarker for STAD, LGG and SKCM, while HAMP is a common biomarker for LUAD, KIRC and SKCM.

LGALS9 encodes galectin 9, which has been demonstrated to be overexpressed in all KIRC tissues and was isolated as a novel immunotherapy target from a cDNA library (39). In glioma, the expression level of LGALS9 can be scored by the immunoreactive score (IRS), which is correlated with the WHO grade, although LGALS9 expression is lower in LGG than in grade IV glioma (40). Furthermore, research has indicated that primary melanoma lesions and melanocytic nevi have high expression of LGALS9, but the minimal expression is found in metastatic melanoma lesions due to the tumor-suppressor function of LGALS9, which inhibits metastatic progression (40, 41).

FCER1G, which encodes the Fc fragment of the IgE receptor Ig, is involved in allergic reactions. Previously, the correlation between KIRC progression and prognosis and FCER1G expression was identified and validated, providing a new immune-related pathway to improve prognosis (42). Although research has reported that FCER1G genes are expressed at higher levels in pilocytic astrocytomas than in LGG, there are no reports of FCER1G expression levels in LGG and SKCM (43).

HAMP, which shows biased expression in the liver and heart, encodes a protein that maintains iron homeostasis primarily through the regulation of iron storage in macrophages and absorption in the intestine (44, 45). The current study confirms that high serum hepcidin is linked to aggressiveness, progression and prognosis in KIRC, making it a potential biomarker to monitor tumor development (46). Similarly, hepcidin concentration is high in the serum of patients with non-small cell lung cancer and is closely associated with tumor clinical stage and lymph node metastasis (47). However, there is no evidence to indicate a correlation between HAMP and SKCM. The above conclusions are all consistent with our results.

Although TMEM149 and EVI2B have been identified by RNA-seq in a variety of different tissues, it is difficult to find any studies about TMEM149 in KIRC, LGG and SKCM or about EVI2B in STAD, LGG and SKCM (44, 48). Thus, according to our results and consistent with the research conclusions reported, TMEM149 and EVI2B are required further study with corresponding tumor types to find better prognostic biomarkers. In addition, we found 74 other immune-related genes with a significant prognostic role for at least one tumor type, some of which have been reported by previous studies (49–52). Overall, these results suggest new possibilities for immune-related prognostic biomarkers, but further experimental and functional studies urgently need to be carried out to validate their predictive roles.

In particular, the increasing evidence of immunotherapy as a major tool for the management of cancer patients points toward the need for testing stromal and immune scores in patients undergoing immunotherapy in search for gene signatures which may better characterize clinical response to immunotherapy (53, 54). Thus, the stromal and immune scores can act as new indicators to broaden the emerging therapeutic landscape in the immunotherapy field.

## Conclusions

In conclusion, the prognosis of six malignant tumor types with poor prognosis could be effectively predicted from the tumor microenvironment as described by immune or stromal scores. Furthermore, a list of immune-related genes was screened as potential biomarkers to predict prognosis for one or more tumor types. Common biomarkers associated with multiple tumor types could contribute to understanding the potential relationships and shared mechanisms among different tumor types. Finally, further functional study of these genes is essential to advance the development of immune-related biomarkers for tumor development and prognosis prediction.

## Data Availability Statement

All datasets generated for this study are included in the article/Supplementary Material.

## Author Contributions

YZ: conceptualization and supervision. YL: data curation and writing—original draft. HZ: formal analysis and software. WF: funding acquisition and writing—review & editing. XZ and WL: investigation. JZ: methodology and resources. GH: project administration. WY: visualization. All authors contributed to the article and approved the submitted version.

## Conflict of Interest

The authors declare that the research was conducted in the absence of any commercial or financial relationships that could be construed as a potential conflict of interest.
