# Supplementary material for: Identification of Immune-Related Prognostic Biomarkers Based on the Tumor Microenvironment in 20 Malignant Tumor Types With Poor Prognosis
Source: Front Oncol. 2020 Jul 31;10:1008. doi: 10.3389/fonc.2020.01008 (PMC7438715; doi:10.3389/fonc.2020.01008)
Supplement: Supplementary file 1 [file Data_Sheet_1.docx]

Supplementary Material

Table S1. DEGs for upregulated and downregulated genes with immune scores.

| **Group** | **Combinations of tumor** | **Total** | **Gene symbol** |
| --- | --- | --- | --- |
| Upregulated genes | BRCA, LUAD, KIRC, LGG and SKCM | 54 | C16orf54 |
|  |  |  | HLA-DRB5 |
|  |  |  | SPN |
|  |  |  | CCL22 |
|  |  |  | EVI2A |
|  |  |  | GSDMA |
|  |  |  | RARRES3 |
|  |  |  | SERPING1 |
|  |  |  | HVCN1 |
|  |  |  | XAF1 |
|  |  |  | ARHGAP25 |
|  |  |  | OLR1 |
|  |  |  | NCF1 |
|  |  |  | BCL2A1 |
|  |  |  | SLA2 |
|  |  |  | LILRB3 |
|  |  |  | SLC37A2 |
|  |  |  | EVI2B |
|  |  |  | C1orf38 |
|  |  |  | ITGAX |
|  |  |  | IL10RA |
|  |  |  | GPR120 |
|  |  |  | FCER1G |
|  |  |  | HAMP |
|  |  |  | CFD |
|  |  |  | ITGAL |
|  |  |  | IL2RG |
|  |  |  | ZAP70 |
|  |  |  | RAC2 |
|  |  |  | CFH |
|  |  |  | LAG3 |
|  |  |  | HLA-DQB1 |
|  |  |  | HLA-DPA1 |
|  |  |  | TNFSF12-TNFSF13 |
|  |  |  | HLA-DRB6 |
|  |  |  | ITGAM |
|  |  |  | ASGR2 |
|  |  |  | IL2RA |
|  |  |  | TMEM149 |
|  |  |  | SYK |
|  |  |  | LPAR5 |
|  |  |  | SASH3 |
|  |  |  | LGALS9 |
|  |  |  | CCL21 |
|  |  |  | RSAD2 |
|  |  |  | HLA-DRB1 |
|  |  |  | MYO1F |
|  |  |  | CFB |
|  |  |  | MYO1G |
|  |  |  | CSF2RA |
|  |  |  | STAC3 |
|  |  |  | CLIC2 |
|  |  |  | FAM70A |
|  |  |  | IL2RB |
|  | BRCA, LUAD and SKCM | 16 | NTRK1 |
|  |  |  | UBE2L6 |
|  |  |  | BFSP2 |
|  |  |  | CLIC5 |
|  |  |  | LGALS2 |
|  |  |  | PM20D1 |
|  |  |  | PIPOX |
|  |  |  | XPNPEP2 |
|  |  |  | SV2B |
|  |  |  | DPT |
|  |  |  | S1PR1 |
|  |  |  | DAZL |
|  |  |  | PAPLN |
|  |  |  | CCL25 |
|  |  |  | CFP |
|  |  |  | BCAT1 |
|  | KIRC and LGG | 4 | TMC6 |
|  |  |  | BMF |
|  |  |  | ATP8B3 |
|  |  |  | PLTP |
| Downregulated genes | KIRC and LGG | 10 | STXBP6 |
|  |  |  | ADCY5 |
|  |  |  | TMEM35 |
|  |  |  | HMGCLL1 |
|  |  |  | SGSM1 |
|  |  |  | ELAVL2 |
|  |  |  | PCP4 |
|  |  |  | TPPP |
|  |  |  | PEG10 |
|  |  |  | BEX5 |

Table S2. DEGs for upregulated and downregulated genes with stromal scores.

| **Group** | **Combinations of tumor** | **Total** | **Gene symbol** | |
| --- | --- | --- | --- | --- |
| Upregulated genes | STAD, LGG and SKCM | 115 | ITGA5 |  |
|  |  |  | ADAM12 |  |
|  |  |  | C16orf54 |  |
|  |  |  | HLA-DRB5 |  |
|  |  |  | SPN |  |
|  |  |  | PCOLCE |  |
|  |  |  | CCL22 |  |
|  |  |  | FNDC1 |  |
|  |  |  | SERPING1 |  |
|  |  |  | EDNRA |  |
|  |  |  | RHOH |  |
|  |  |  | HVCN1 |  |
|  |  |  | MRGPRF |  |
|  |  |  | LTBP1 |  |
|  |  |  | FN1 |  |
|  |  |  | FAIM3 |  |
|  |  |  | SIX2 |  |
|  |  |  | P2RY8 |  |
|  |  |  | ARHGAP25 |  |
|  |  |  | GALNTL2 |  |
|  |  |  | OLR1 |  |
|  |  |  | NCF1 |  |
|  |  |  | SIGLEC1 |  |
|  |  |  | MGP |  |
|  |  |  | TGFB1 |  |
|  |  |  | CHST2 |  |
|  |  |  | TIMP1 |  |
|  |  |  | CPZ |  |
|  |  |  | ABI3 |  |
|  |  |  | IGFBP5 |  |
|  |  |  | FPR1 |  |
|  |  |  | PTPRC |  |
|  |  |  | ARHGDIB |  |
|  |  |  | COL4A2 |  |
|  |  |  | C1S |  |
|  |  |  | AXL |  |
|  |  |  | CYSLTR1 |  |
|  |  |  | SRPX2 |  |
|  |  |  | PLA2G2D |  |
|  |  |  | STAB1 |  |
|  |  |  | PDLIM4 |  |
|  |  |  | CLEC7A |  |
|  |  |  | PALMD |  |
|  |  |  | EVI2B |  |
|  |  |  | C1orf38 |  |
|  |  |  | THBS1 |  |
|  |  |  | LHFP |  |
|  |  |  | P2RY10 |  |
|  |  |  | SLC15A3 |  |
|  |  |  | ITGAX |  |
|  |  |  | C1orf54 |  |
|  |  |  | IL10RA |  |
|  |  |  | COL4A1 |  |
|  |  |  | LOX |  |
|  |  |  | ELTD1 |  |
|  |  |  | FCER1G |  |
|  |  |  | IGFBP3 |  |
|  |  |  | PLVAP |  |
|  |  |  | COL11A1 |  |
|  |  |  | CCDC152 |  |
|  |  |  | GJA5 |  |
|  |  |  | GGT5 |  |
|  |  |  | CFD |  |
|  |  |  | ITGAL |  |
|  |  |  | CH25H |  |
|  |  |  | KLRB1 |  |
|  |  |  | LAMA2 |  |
|  |  |  | C1R |  |
|  |  |  | FPR3 |  |
|  |  |  | TRIM22 |  |
|  |  |  | GJA1 |  |
|  |  |  | TGM2 |  |
|  |  |  | COL15A1 |  |
|  |  |  | CFH |  |
|  |  |  | IGFBP7 |  |
|  |  |  | ITGA1 |  |
|  |  |  | APLNR |  |
|  |  |  | HLA-DQB1 |  |
|  |  |  | TSHZ2 |  |
|  |  |  | CTF1 |  |
|  |  |  | LTBP2 |  |
|  |  |  | STEAP4 |  |
|  |  |  | STC1 |  |
|  |  |  | HLA-DPA1 |  |
|  |  |  | DES |  |
|  |  |  | HLA-DMB |  |
|  |  |  | H19 |  |
|  |  |  | MRC2 |  |
|  |  |  | SRGN |  |
|  |  |  | C10orf10 |  |
|  |  |  | HLA-DRB6 |  |
|  |  |  | ITGA4 |  |
|  |  |  | ITGAM |  |
|  |  |  | PDPN |  |
|  |  |  | IL2RA |  |
|  |  |  | FAM198B |  |
|  |  |  | RARRES2 |  |
|  |  |  | PAMR1 |  |
|  |  |  | SASH3 |  |
|  |  |  | COL14A1 |  |
|  |  |  | CCL21 |  |
|  |  |  | VCAM1 |  |
|  |  |  | RSAD2 |  |
|  |  |  | TGFBR2 |  |
|  |  |  | GLIPR1 |  |
|  |  |  | MYO1F |  |
|  |  |  | ITGA9 |  |
|  |  |  | MYO1G |  |
|  |  |  | CSF2RA |  |
|  |  |  | PTPLAD2 |  |
|  |  |  | PI16 |  |
|  |  |  | REM1 |  |
|  |  |  | MFNG |  |
|  |  |  | CLIC2 |  |
|  |  |  | IL2RB |  |
|  | STAD and LGG | 23 | IFI16 |  |
|  |  |  | PLEKHO2 |  |
|  |  |  | BCL2A1 |  |
|  |  |  | OGFRL1 |  |
|  |  |  | CLIC4 |  |
|  |  |  | GAL3ST4 |  |
|  |  |  | SYDE1 |  |
|  |  |  | LAMB2 |  |
|  |  |  | TGFBR3 |  |
|  |  |  | GJC1 |  |
|  |  |  | LMCD1 |  |
|  |  |  | ACTN1 |  |
|  |  |  | WLS |  |
|  |  |  | LGALS1 |  |
|  |  |  | COPZ2 |  |
|  |  |  | LAMB1 |  |
|  |  |  | RIN3 |  |
|  |  |  | CAV1 |  |
|  |  |  | HOXA4 |  |
|  |  |  | MATN2 |  |
|  |  |  | MAP7D3 |  |
|  |  |  | FBXO32 |  |
|  |  |  | HLX |  |
| Downregulated genes | STAD, LGG and SKCM | 1 | TMEM97 |  |
|  | STAD and LGG | 7 | KRTCAP3 |  |
|  |  |  | ACAT2 |  |
|  |  |  | TOX3 |  |
|  |  |  | LRRC26 |  |
|  |  |  | SPC25 |  |
|  |  |  | NMU |  |
|  |  |  | PRSS3 |  |
|  |  |  |  |  |

Table S3. GO enrichment analysis of DEGs detected 54 for immune scores group and 116 for stromal scores group.

| **Group** | **Category** | **Term** | **Count** | **%** | **PValue** | **FDR** |
| --- | --- | --- | --- | --- | --- | --- |
| **Immune** | GOTERM_BP_FAT | GO:0006955~immune response | 24 | 44.44 | 4.62E-19 | 6.74E-16 |
| **scores** | GOTERM_BP_FAT | GO:0002684~positive regulation of immune system process | 14 | 25.93 | 2.55E-13 | 3.73E-10 |
|  | GOTERM_BP_FAT | GO:0050778~positive regulation of immune response | 10 | 18.52 | 6.02E-10 | 8.78E-07 |
|  | GOTERM_BP_FAT | GO:0006952~defense response | 15 | 27.78 | 3.21E-09 | 4.68E-06 |
|  | GOTERM_BP_FAT | GO:0051249~regulation of lymphocyte activation | 9 | 16.67 | 1.78E-08 | 2.60E-05 |
|  | GOTERM_BP_FAT | GO:0048584~positive regulation of response to stimulus | 10 | 18.52 | 4.32E-08 | 6.31E-05 |
|  | GOTERM_BP_FAT | GO:0002694~regulation of leukocyte activation | 9 | 16.67 | 4.37E-08 | 6.37E-05 |
|  | GOTERM_BP_FAT | GO:0050865~regulation of cell activation | 9 | 16.67 | 6.59E-08 | 9.61E-05 |
|  | GOTERM_BP_FAT | GO:0002252~immune effector process | 8 | 14.81 | 1.91E-07 | 2.79E-04 |
|  | GOTERM_BP_FAT | GO:0002253~activation of immune response | 7 | 12.96 | 4.75E-07 | 6.93E-04 |
|  | GOTERM_BP_FAT | GO:0051251~positive regulation of lymphocyte activation | 7 | 12.96 | 5.72E-07 | 8.35E-04 |
|  | GOTERM_BP_FAT | GO:0002697~regulation of immune effector process | 7 | 12.96 | 7.27E-07 | 0.0011 |
|  | GOTERM_BP_FAT | GO:0002696~positive regulation of leukocyte activation | 7 | 12.96 | 9.67E-07 | 0.0014 |
|  | GOTERM_BP_FAT | GO:0050867~positive regulation of cell activation | 7 | 12.96 | 1.27E-06 | 0.0019 |
|  | GOTERM_BP_FAT | GO:0002703~regulation of leukocyte mediated immunity | 6 | 11.11 | 1.33E-06 | 0.0019 |
|  | GOTERM_BP_FAT | GO:0050863~regulation of T cell activation | 7 | 12.96 | 1.73E-06 | 0.0025 |
|  | GOTERM_BP_FAT | GO:0045582~positive regulation of T cell differentiation | 5 | 9.26 | 2.98E-06 | 0.0044 |
|  | GOTERM_BP_FAT | GO:0002504~antigen processing and presentation of peptide or polysaccharide antigen via MHC class II | 5 | 9.26 | 3.39E-06 | 0.0049 |
|  | GOTERM_BP_FAT | GO:0050870~positive regulation of T cell activation | 6 | 11.11 | 3.99E-06 | 0.0058 |
|  | GOTERM_BP_FAT | GO:0045621~positive regulation of lymphocyte differentiation | 5 | 9.26 | 4.31E-06 | 0.0063 |
|  | GOTERM_BP_FAT | GO:0006954~inflammatory response | 9 | 16.67 | 7.09E-06 | 0.0103 |
|  | GOTERM_BP_FAT | GO:0050864~regulation of B cell activation | 5 | 9.26 | 1.98E-05 | 0.0289 |
|  | GOTERM_BP_FAT | GO:0045580~regulation of T cell differentiation | 5 | 9.26 | 1.98E-05 | 0.0289 |
|  | GOTERM_BP_FAT | GO:0002706~regulation of lymphocyte mediated immunity | 5 | 9.26 | 2.49E-05 | 0.0364 |
|  | GOTERM_BP_FAT | GO:0050671~positive regulation of lymphocyte proliferation | 5 | 9.26 | 2.68E-05 | 0.0391 |
|  | GOTERM_BP_FAT | GO:0070665~positive regulation of leukocyte proliferation | 5 | 9.26 | 2.88E-05 | 0.0420 |
|  | GOTERM_BP_FAT | GO:0032946~positive regulation of mononuclear cell proliferation | 5 | 9.26 | 2.88E-05 | 0.0420 |
|  | GOTERM_BP_FAT | GO:0046638~positive regulation of alpha-beta T cell differentiation | 4 | 7.41 | 3.28E-05 | 0.0479 |
|  | GOTERM_CC_FAT | GO:0009897~external side of plasma membrane | 8 | 14.81 | 2.25E-06 | 0.0024 |
|  | GOTERM_CC_FAT | GO:0044459~plasma membrane part | 22 | 40.74 | 7.03E-06 | 0.0074 |
|  | GOTERM_CC_FAT | GO:0009986~cell surface | 9 | 16.67 | 2.94E-05 | 0.0310 |
|  | GOTERM_CC_FAT | GO:0005887~integral to plasma membrane | 15 | 27.78 | 4.63E-05 | 0.0489 |
|  | GOTERM_MF_FAT | GO:0004896~cytokine receptor activity | 5 | 9.26 | 1.91E-05 | 0.0225 |
|  | GOTERM_MF_FAT | GO:0019976~interleukin-2 binding | 3 | 5.56 | 2.50E-05 | 0.0294 |
|  | GOTERM_MF_FAT | GO:0004911~interleukin-2 receptor activity | 3 | 5.56 | 2.50E-05 | 0.0294 |
| **Stromal** | GOTERM_BP_FAT | GO:0006952~defense response | 26 | 22.41 | 1.62E-13 | 2.63E-10 |
| **scores** | GOTERM_BP_FAT | GO:0006954~inflammatory response | 20 | 17.24 | 3.83E-13 | 6.22E-10 |
|  | GOTERM_BP_FAT | GO:0006955~immune response | 26 | 22.41 | 2.11E-12 | 3.42E-09 |
|  | GOTERM_BP_FAT | GO:0009611~response to wounding | 23 | 19.83 | 4.09E-12 | 6.64E-09 |
|  | GOTERM_BP_FAT | GO:0007155~cell adhesion | 24 | 20.69 | 1.38E-10 | 2.24E-07 |
|  | GOTERM_BP_FAT | GO:0022610~biological adhesion | 24 | 20.69 | 1.42E-10 | 2.30E-07 |
|  | GOTERM_BP_FAT | GO:0002684~positive regulation of immune system process | 15 | 12.93 | 5.97E-10 | 9.69E-07 |
|  | GOTERM_BP_FAT | GO:0007159~leukocyte adhesion | 7 | 6.03 | 2.62E-08 | 4.26E-05 |
|  | GOTERM_BP_FAT | GO:0048584~positive regulation of response to stimulus | 13 | 11.21 | 5.62E-08 | 9.12E-05 |
|  | GOTERM_BP_FAT | GO:0016337~cell-cell adhesion | 13 | 11.21 | 3.08E-07 | 5.00E-04 |
|  | GOTERM_BP_FAT | GO:0007166~cell surface receptor linked signal transduction | 32 | 27.59 | 6.50E-07 | 0.0011 |
|  | GOTERM_BP_FAT | GO:0050867~positive regulation of cell activation | 9 | 7.76 | 7.69E-07 | 0.0012 |
|  | GOTERM_BP_FAT | GO:0032101~regulation of response to external stimulus | 10 | 8.62 | 1.17E-06 | 0.0019 |
|  | GOTERM_BP_FAT | GO:0002252~immune effector process | 9 | 7.76 | 3.20E-06 | 0.0052 |
|  | GOTERM_BP_FAT | GO:0045321~leukocyte activation | 11 | 9.48 | 5.03E-06 | 0.0082 |
|  | GOTERM_BP_FAT | GO:0050778~positive regulation of immune response | 9 | 7.76 | 5.76E-06 | 0.0094 |
|  | GOTERM_BP_FAT | GO:0002696~positive regulation of leukocyte activation | 8 | 6.90 | 7.01E-06 | 0.0114 |
|  | GOTERM_BP_FAT | GO:0007229~integrin-mediated signaling pathway | 7 | 6.03 | 7.28E-06 | 0.0118 |
|  | GOTERM_BP_FAT | GO:0006928~cell motion | 14 | 12.07 | 1.65E-05 | 0.0267 |
|  | GOTERM_BP_FAT | GO:0001775~cell activation | 11 | 9.48 | 2.23E-05 | 0.0362 |
|  | GOTERM_BP_FAT | GO:0050865~regulation of cell activation | 9 | 7.76 | 2.28E-05 | 0.0370 |
|  | GOTERM_CC_FAT | GO:0005576~extracellular region | 44 | 37.93 | 3.06E-10 | 3.70E-07 |
|  | GOTERM_CC_FAT | GO:0009897~external side of plasma membrane | 14 | 12.07 | 1.10E-09 | 1.33E-06 |
|  | GOTERM_CC_FAT | GO:0044421~extracellular region part | 29 | 25.00 | 1.58E-09 | 1.92E-06 |
|  | GOTERM_CC_FAT | GO:0008305~integrin complex | 7 | 6.03 | 1.02E-07 | 1.24E-04 |
|  | GOTERM_CC_FAT | GO:0031012~extracellular matrix | 16 | 13.79 | 1.17E-07 | 1.42E-04 |
|  | GOTERM_CC_FAT | GO:0009986~cell surface | 16 | 13.79 | 1.31E-07 | 1.59E-04 |
|  | GOTERM_CC_FAT | GO:0005578~proteinaceous extracellular matrix | 15 | 12.93 | 2.99E-07 | 3.61E-04 |
|  | GOTERM_CC_FAT | GO:0031093~platelet alpha granule lumen | 7 | 6.03 | 8.95E-07 | 0.0011 |
|  | GOTERM_CC_FAT | GO:0060205~cytoplasmic membrane-bounded vesicle lumen | 7 | 6.03 | 1.38E-06 | 0.0017 |
|  | GOTERM_CC_FAT | GO:0031983~vesicle lumen | 7 | 6.03 | 1.81E-06 | 0.0022 |
|  | GOTERM_CC_FAT | GO:0044420~extracellular matrix part | 9 | 7.76 | 4.80E-06 | 0.0058 |
|  | GOTERM_CC_FAT | GO:0031091~platelet alpha granule | 7 | 6.03 | 5.86E-06 | 0.0071 |
|  | GOTERM_CC_FAT | GO:0005581~collagen | 6 | 5.17 | 8.66E-06 | 0.0105 |
|  | GOTERM_CC_FAT | GO:0005615~extracellular space | 19 | 16.38 | 9.22E-06 | 0.0112 |
|  | GOTERM_CC_FAT | GO:0044459~plasma membrane part | 37 | 31.90 | 1.37E-05 | 0.0166 |
|  | GOTERM_CC_FAT | GO:0005886~plasma membrane | 52 | 44.83 | 2.13E-05 | 0.0257 |
|  | GOTERM_MF_FAT | GO:0019838~growth factor binding | 11 | 9.48 | 2.59E-09 | 0.0000 |
|  | GOTERM_MF_FAT | GO:0005201~extracellular matrix structural constituent | 7 | 6.03 | 2.83E-05 | 0.0366 |
|  | GOTERM_MF_FAT | GO:0030246~carbohydrate binding | 12 | 10.34 | 3.21E-05 | 0.0417 |

Table S4. Correlation of DEGs for stromal score group with prognosis in associated cancers by Kaplan-Meier survival curves from TCGA.

|  | **DEGs** | **p (STAD)** | **p (LGG)** | **p (SKCM)** |
| --- | --- | --- | --- | --- |
| 1 | ABI3 | - | < 0.0001 | 0.035 |
| 2 | ADAM12 | 0.013 | < 0.0001 | - |
| 3 | APLNR | - | 0.04 | - |
| 4 | ARHGAP25 | - | < 0.0001 | 0.0024 |
| 5 | ARHGDIB | - | 0.003 | 0.0047 |
| 6 | AXL | 0.0095 | 0.0001 | 0.0019 |
| 7 | C10orf10 | 0.01 | < 0.0001 | - |
| 8 | C16orf54 | - | < 0.0001 | 0.0003 |
| 9 | C1orf38 | - | 0.032 | - |
| 10 | C1orf54 | - | < 0.0001 | 0.0022 |
| 11 | C1R | - | < 0.0001 | 0.0046 |
| 12 | C1S | - | < 0.0001 | 0.0065 |
| 13 | CCDC152 | 0.014 | < 0.0001 | 0.03 |
| 14 | CCL22 | - | 0.00055 | - |
| 15 | CFD | - | 0.0035 | - |
| 16 | CFH | - | < 0.0001 | - |
| 17 | CHST2 | - | < 0.0001 | - |
| 18 | CLEC7A | - | 0.00022 | 0.00043 |
| 19 | CLIC2 | - | - | 0.00028 |
| 20 | COL11A1 | - | 0.011 | - |
| 21 | COL14A1 | - | 0.035 | - |
| 22 | COL15A1 | - | 0.0017 | - |
| 23 | COL4A1 | - | <0.0001 | - |
| 24 | COL4A2 | - | < 0.0001 | - |
| 25 | CSF2RA | - | 0.0086 | - |
| 26 | CTF1 | 0.026 | < 0.0001 | - |
| 27 | CYSLTR1 | - | - | <0.0001 |
| 28 | DES | 0.014 | < 0.0001 | - |
| 29 | ELTD1 | 0.034 | 0.02 | - |
| 30 | EVI2B | 0.04 | < 0.0001 | 0.00011 |
| 31 | FAIM3 | - | 0.00018 | - |
| 32 | FAM198B | - | 0.043 | - |
| 33 | FCER1G | - | < 0.0001 | 0.0017 |
| 34 | FN1 | 0.0076 | < 0.0001 | - |
| 35 | FNDC1 | 0.027 | - | - |
| 36 | FPR1 | - | 0.015 | 0.0011 |
| 37 | FPR3 | - | 0.0016 | 0.031 |
| 38 | GALNTL2 | 0.024 | - | - |
| 39 | GGT5 | 0.0053 | < 0.0001 | - |
| 40 | GJA1 | 0.0057 | < 0.0001 | - |
| 41 | GJA5 | - | 0.013 | - |
| 42 | GLIPR1 | 0.017 | < 0.0001 | 0.016 |
| 43 | H19 | 0.011 | < 0.0001 | - |
| 44 | HLA-DMB | - | < 0.0001 | <0.0001 |
| 45 | HLA-DPA1 | - | < 0.0001 | <0.0001 |
| 46 | HLA-DQB1 | - | < 0.0001 | <0.0001 |
| 47 | HLA-DRB5 | - | < 0.0001 | <0.0001 |
| 48 | HLA-DRB6 | - | 0.00074 | <0.0001 |
| 49 | HVCN1 | - | < 0.0001 | 0.031 |
| 50 | IGFBP3 | - | < 0.0001 | - |
| 51 | IGFBP5 | 0.021 | < 0.0001 | - |
| 52 | IGFBP7 | 0.0068 | 0.0011 | - |
| 53 | IL10RA | - | 0.017 | 0.00019 |
| 54 | IL2RA | - | - | <0.0001 |
| 55 | IL2RB | - | 0.00047 | 0.007 |
| 56 | ITGA1 | - | < 0.0001 | 0.0053 |
| 57 | ITGA4 | - | 0.00011 | - |
| 58 | ITGA5 | - | < 0.0001 | - |
| 59 | ITGA9 | 0.017 | - | - |
| 60 | ITGAL | - | < 0.0001 | 0.0014 |
| 61 | ITGAM | - | 0.0089 | 0.0002 |
| 62 | ITGAX | - | < 0.0001 | 0.0025 |
| 63 | KLRB1 | - | < 0.0001 | 0.0021 |
| 64 | LAMA2 | 0.017 | < 0.0001 | - |
| 65 | LHFP | 0.043 | < 0.0001 | - |
| 66 | LOX | 0.0041 | < 0.0001 | - |
| 67 | LTBP1 | - | < 0.0001 | - |
| 68 | MFNG | - | < 0.0001 | 0.009 |
| 69 | MGP | 0.027 | 0.00048 | - |
| 70 | MRC2 | - | < 0.0001 | - |
| 71 | MRGPRF | 0.028 | 0.0097 | - |
| 72 | MYO1F | - | 0.0014 | 0.01 |
| 73 | MYO1G | - | 0.00056 | 0.00059 |
| 74 | NCF1 | - | < 0.0001 | <0.0001 |
| 75 | OLR1 | - | < 0.0001 | 0.002 |
| 76 | P2RY10 | - | 0.00092 | 0.0034 |
| 77 | P2RY8 | - | 0.00044 | 0.0025 |
| 78 | PALMD | - | < 0.0001 | - |
| 79 | PAMR1 | 0.029 | 0.00036 | - |
| 80 | PCOLCE | - | < 0.0001 | - |
| 81 | PDLIM4 | 0.029 | < 0.0001 | - |
| 82 | PDPN | - | < 0.0001 | - |
| 83 | PLA2G2D | - | - | 0.00042 |
| 84 | PTPLAD2 | - | < 0.0001 | - |
| 85 | PTPRC | - | < 0.0001 | <0.0001 |
| 86 | RARRES2 | - | < 0.0001 | - |
| 87 | REM1 | 0.014 | - | 0.0068 |
| 88 | RHOH | - | < 0.0001 | 0.00039 |
| 89 | RSAD2 | - | < 0.0001 | 0.00012 |
| 90 | SASH3 | - | < 0.0001 | - |
| 91 | SERPING1 | 0.038 | < 0.0001 | 0.00043 |
| 92 | SIGLEC1 | - | < 0.0001 | 0.0031 |
| 93 | SIX2 | - | 0.00094 | - |
| 94 | SLC15A3 | - | < 0.0001 | 0.00036 |
| 95 | SPN | - | < 0.0001 | 0.0011 |
| 96 | SRGN | - | - | 0.00012 |
| 97 | SRPX2 | 0.0013 | < 0.0001 | - |
| 98 | STC1 | 0.019 | 0.0027 | - |
| 99 | STEAP4 | 0.023 | - | - |
| 100 | TGFB1 | - | < 0.0001 | - |
| 101 | TGFBR2 | - | 0.018 | - |
| 102 | TGM2 | - | 0.025 | 0.032 |
| 103 | THBS1 | 0.001 | 0.0026 | - |
| 104 | TIMP1 | - | < 0.0001 | 0.0028 |
| 105 | TRIM22 | - | < 0.0001 | <0.0001 |
| 106 | TSHZ2 | - | 0.00025 | - |
| 107 | VCAM1 | - | 0.0023 | 0.0061 |
| 108 | TMEM97 | - | - | <0.0001 |

-, p > 0.05, no statistical significance.
