# Supplementary material for: Identification of Immune-Related Prognostic Biomarkers Based on the Tumor Microenvironment in 20 Malignant Tumor Types With Poor Prognosis
Source: Front Oncol. 2020 Jul 31;10:1008. doi: 10.3389/fonc.2020.01008 (PMC7438715; doi:10.3389/fonc.2020.01008)
Supplement: Supplementary file 2 [file Data_Sheet_2.docx]

Supplementary Material


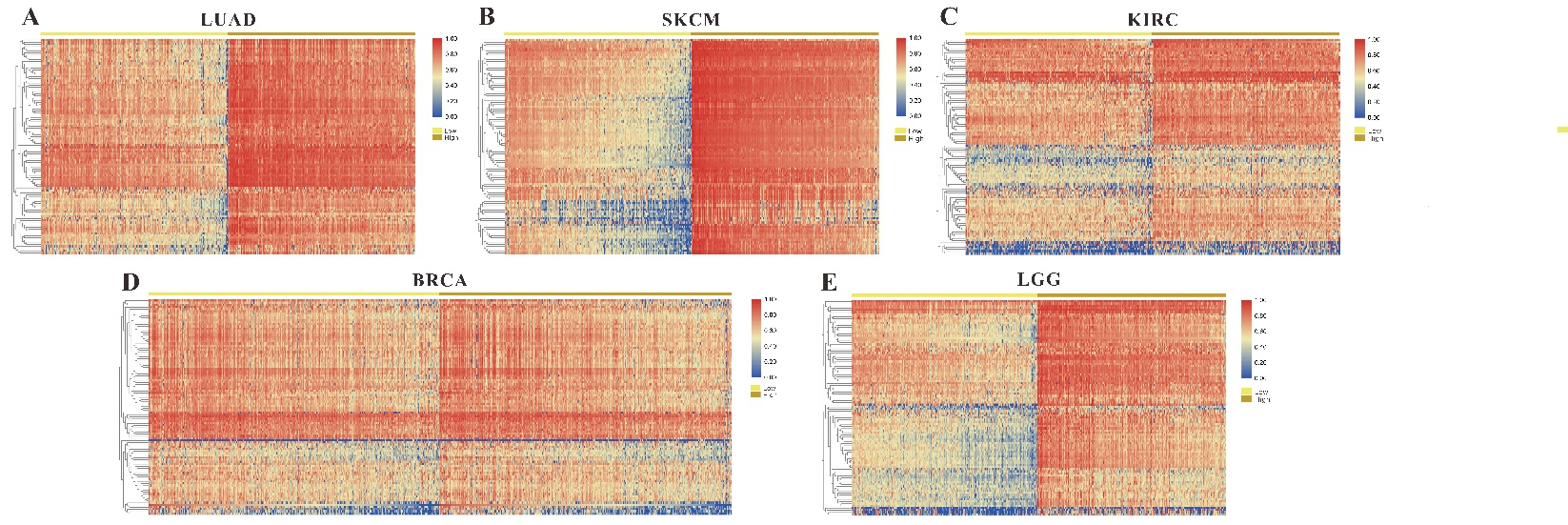


**Figure S1.** The heatmaps of top 100 DEGs by comparing the low score group with high score group of immune scores for LUAD (A), SKCM (B), KIRC (C), BRCA (D) and LGG (E).

**
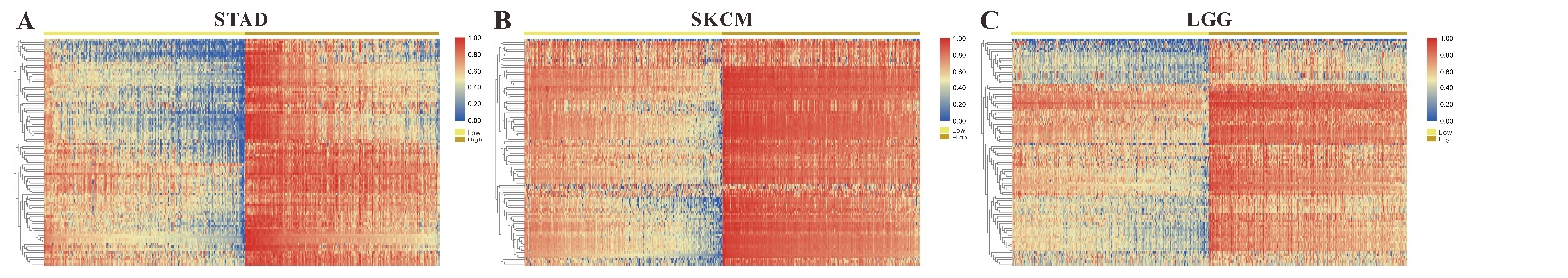
**

**Figure S2.** The heatmaps of top 100 DEGs by comparing the low score group with high score group of stromal scores for STAD (A), SKCM (B), and LGG (C).


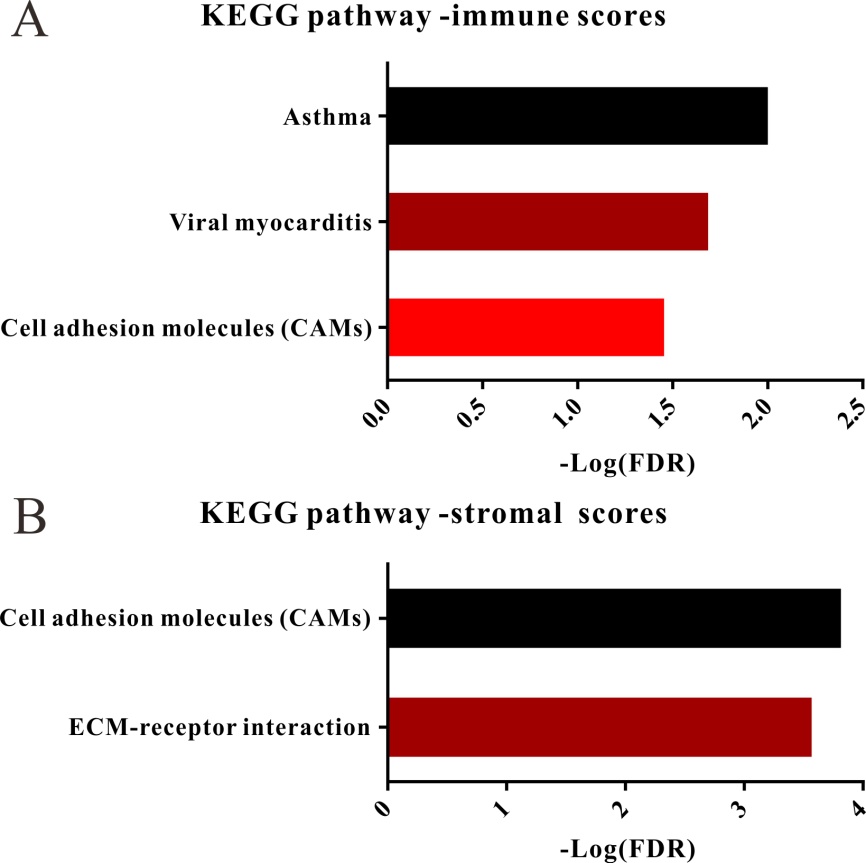
、

**Figure S3.** KEGG pathway analysis for immune and stromal score groups.

**

**

**Figure S4.** The expression levels for genes with statistical significance both in 2 tumor types. * p-Value<0.05.

.
